# Draft Genome Sequence of *Cloacibacterium normanense* NRS-1 Isolated from Municipal Wastewater

**DOI:** 10.1128/genomeA.01397-16

**Published:** 2016-12-15

**Authors:** Nicole R. Gay, Elizabeth Fleming, Julia Oh

**Affiliations:** The Jackson Laboratory for Genomic Medicine, Farmington, Connecticut, USA

## Abstract

*Cloacibacterium normanense* is a Gram-negative bacterium recovered from untreated human wastewater. Given its high abundance in wastewater and its apparent absence in human stool, it may contribute to biological phosphate removal. Here, we perform a whole-genome sequence of *C. normanense* NRS-1(T) and examine particular features of this draft genome.

## GENOME ANNOUNCEMENT

Three strains of a novel bacterium were isolated from untreated human wastewater at a municipal water treatment plant in Norman, Oklahoma, USA (1). These strains were assigned to the novel genus *Cloacibacterium*, all within the species *Cloacibacterium normanense*. *C. normanense* NRS-1 was the designated type strain (1). *C. normanense* is a Gram-negative, nonmotile, yellow-pigmented, rod-shaped, facultatively anaerobic member of the *Flavobactericeae* family (1). Specifically, it belongs to a cluster within the *Bergeyella*-*Chryseobacterium*-*Riemerella* branch that is closely shared by *Riemerella columbina*, *Bergeyella zoohelcum*, and *Riemerella anatipestifer* (1).

Given the pervasiveness of *Flavobacteriaceae* members in aquatic habitats, the ability of some of these members to decompose complex organic molecules, and the high abundance of *C. normanense* in untreated wastewater (estimated counts of 1.4E5 cells/mL and 1.4E4 cells/mL at two respective water treatment plants), *C. normanense* may play a role in phosphate removal (1–4). While PCR techniques did not detect the bacterium in any of the 10 human stool samples tested (1), an undefined species belonging to the *Cloacibacterium* genus has been identified in human skin (5).

DNA was extracted from a *C. normanense* NRS-1 sample procured from ATCC, and a Nextera XT kit was used for library preparation. Whole-genome sequencing was performed by Illumina HiSeq, with a read length of 2 × 150 bp and an average insert size of 205 bp. Adapters were trimmed using Cutadapt (6), poor quality bases were removed using trimBWAstyle.pl, and reads <50 bp after trimming were removed using PRINSEQ-lite (7). Sequencing yielded 6,648,070 paired reads with a 33.03% GC content. SPAdes version 3.7.1 was used to assemble the reads with 494× mean coverage of the resulting contigs (8). QUAST reported a total scaffold length of 2,721,964 bp with a maximum scaffold length of 197,666 bp and an *N*_50_ value of 112,075 bp (9).

The IGS Annotation Engine was used for structural and functional annotation of the sequences (http://ae.igs.umaryland.edu/cgi/index.cgi, reference: 21677861). Manatee was used to view annotations (http://manatee.sourceforge.net). Manatee identified 38 tRNAs, three rRNAs, and 2,579 open reading frames, 88.5% of which are coding regions. The Comprehensive Antibiotic Resistance Database (CARD) recognized resistance genes for kanamycin and streptomycin, confirming positive *in vitro* resistance tests conducted by Allen et al. (10, 11). However, the *in vitro* tests also indicated resistance to erythromycin, which suggests that the *C. normanense* genome contains an uncharacterized erythromycin resistance gene. CARD also suggested resistance to six additional antibiotics not tested by Allen et al.: amikacin, kanamycin A, neomycin, spectinomycin, tobramycin, and viomycin. antiSMASH version 3.0 identified a terpene secondary metabolite cluster, most similar to the carotenoid biosynthetic gene cluster (28% of the genes show similarity) (12). CRISPRfinder identified three questionable clustered regularly interspaced short palindromic repeats (CRISPRs) and one confirmed CRISPR in contig 4088 (13). No intact prophage regions were detected by PHASTER (14, 15). Previously, only 16S sequence data were available for *C. normanense* (1). This draft genome provides a more comprehensive characterization of this bacterial type strain.

### Accession number(s).

This whole-genome shotgun project has been deposited at DDBJ/EMBL/GenBank under the accession number MKGI00000000. The version described in this paper is the first version, MKGI01000000.

## References

[1] Allen TD, Lawson PA, Collins MD, Falsen E, Tanner RS 2006 *Cloacibacterium normanense* gen. nov., sp. nov., a novel bacterium in the family *Flavobacteriaceae* isolated from municipal wastewater. Int J Syst Evol Microbiol 56:1311–1316. doi:10.1099/ijs.0.64218-0. 16738108

[2] Van Ommen Kloeke F, Geesey GG 1999 Localization and identification of populations of phosphatase-active bacterial cells associated with activated sludge. Microb Ecol 38:201–214. doi:10.1007/s002489900170. 10541782

[3] Bernardet JF, Nakagawa Y, Holmes B, Subcommittee on the Taxonomy of Flavobacterium and Cytophaga-Like Bacteria of the International Committee on Systematics of Prokaryotes 2002 Proposed minimal standards for describing new taxa of the family Flavobacteriaceae and emended description of the family. Int J Syst Evol Microbiol 52:1049–1070. doi:10.1099/00207713-52-3-1049. 12054224

[4] Liu Y, Zhang T, Fang HH 2005 Microbial community analysis and performance of a phosphate-removing activated sludge. Bioresour Technol 96:1205–1214. doi:10.1016/j.biortech.2004.11.003. 15734306

[5] Oh J, Freeman AF, NISC Comparative Sequencing Program, Park M, Sokolic R, Candotti F, Holland SM, Segre JA, Kong HH 2013 The altered landscape of the human skin microbiome in patients with primary immunodeficiencies. Genome Res 23:2103–2114. doi:10.1101/gr.159467.113. 24170601 PMC3847779

[6] Martin M 2011 Cutadapt removes adapter sequences from high-throughput sequencing reads. EMBnet J 17:10–12. doi:10.14806/ej.17.1.200.

[7] Schmieder R, Edwards R 2011 Quality control and preprocessing of metagenomic datasets. BioInformatics 27:863–864. doi:10.1093/bioinformatics/btr026. 21278185 PMC3051327

[8] Bankevich A, Nurk S, Antipov D, Gurevich AA, Dvorkin M, Kulikov AS, Lesin VM, Nikolenko SI, Pham S, Prjibelski AD, Pyshkin AV, Sirotkin AV, Vyahhi N, Tesler N, Alekseyev MA, Pevzner PA 2012 SPAdes: a new genome assembly algorithm and its applications to single-cell sequencing. J Comput Biol 19:455–477. doi:10.1089/cmb.2012.0021. 22506599 PMC3342519

[9] Gurevich A, Saveliev V, Vyahhi N, Tesler G 2013 QUAST: quality assessment tool for genome assemblies. Bioinformatics 29:1072–1075. doi:10.1093/bioinformatics/btt086. 23422339 PMC3624806

[10] McArthur AG, Waglechner N, Nizam F, Yan A, Azad MA, Baylay AJ, Bhullar K, Canova MJ, De Pascale G, Ejim L, Kalan L, King AM, Koteva K, Morar M, Mulvey MR, O’Brien JS, Pawlowski AC, Piddock LJ, Spanogiannopoulos P, Sutherland AD, Tang I, Taylor PL, Thaker M, Wang W, Yan M, Yu T, Wright GD 2013 The Comprehensive Antibiotic Resistance Database. Antimicrob Agents Chemother 57:3348–3357. doi:10.1128/AAC.00419-13. 23650175 PMC3697360

[11] McArthur AG, Wright GD 2015 Bioinformatics of antimicrobial resistance in the age of molecular epidemiology. Curr Opin Microbiol 27:45–50. doi:10.1016/j.mib.2015.07.004. 26241506

[12] Weber T, Blin K, Duddela S, Krug D, Kim HU, Bruccoleri R, Lee SY, Fischbach MA, Müller R, Wohlleben W, Breitling R, Takano E, Medema MH 2015 antiSMASH 3.0—a comprehensive resource for the genome mining of biosynthetic gene clusters. Nucleic Acids Res 43:W237–W243. doi:10.1093/nar/gkv437. 25948579 PMC4489286

[13] Grissa I, Vergnaud G, Pourcel C 2007 CRISPRFinder: a web tool to identify clustered regularly interspaced short palindromic repeats. Nucleic Acids Res 35:W52–W57. doi:10.1093/nar/gkm360. 17537822 PMC1933234

[14] Arndt D, Grant JR, Marcu A, Sajed T, Pon A, Liang Y, Wishart DS 2016 PHASTER: a better, faster version of the PHAST phage search tool. Nucleic Acids Res 44:W16–W21 doi:10.1093/nar/gkw387. 27141966 PMC4987931

[15] Zhou Y, Liang Y, Lynch KH, Dennis JJ, Wishart DS 2011 PHAST: a fast phage search tool. Nucleic Acids Res 39:W347–W352. doi:10.1093/nar/gkr485. 21672955 PMC3125810

